# Association of *PARP1* polymorphisms with response to chemotherapy in patients with high‐risk neuroblastoma

**DOI:** 10.1111/jcmm.15058

**Published:** 2020-02-27

**Authors:** Marianna Avitabile, Vito Alessandro Lasorsa, Sueva Cantalupo, Antonella Cardinale, Flora Cimmino, Annalaura Montella, Dalila Capasso, Riccardo Haupt, Loredana Amoroso, Alberto Garaventa, Alessandro Quattrone, Maria Valeria Corrias, Achille Iolascon, Mario Capasso

**Affiliations:** ^1^ Dipartimento di Medicina Molecolare e Biotecnologie Mediche Università degli Studi di Napoli Federico II Naples Italy; ^2^ CEINGE Biotecnologie Avanzate Naples Italy; ^3^ IRCCS SDN Naples Italy; ^4^ UOS Epidemiology, Biostatistics and Committees Genova Italy; ^5^ Department of Pediatric Oncology IRCCS Istituto Giannina Gaslini Genova Italy; ^6^ Laboratory of Translational Genomics Centre for Integrative Biology University of Trento Trento Italy; ^7^ Laboratory of Experimental Therapy in Oncology IRCCS Istituto Giannina Gaslini Genova Italy

**Keywords:** chemotherapy, neuroblastoma, oncology, PARP1, pharmacogenomics, SNP

## Abstract

The genetic aetiology and the molecular mechanisms that characterize high‐risk neuroblastoma are still little understood. The majority of high‐risk neuroblastoma patients do not take advantage of current induction therapy. So far, one of the main reasons liable for cancer therapeutic failure is the acquisition of resistance to cytotoxic anticancer drugs, because of the DNA repair system of tumour cells. *PARP1* is one of the main DNA damage sensors involved in the DNA repair system and genomic stability. We observed that high *PARP1* mRNA level is associated with unfavourable prognosis in 3 public gene expression NB patients’ datasets and in 20 neuroblastomas analysed by qRT‐PCR. Among 4983 SNPs in *PARP1*, we selected two potential functional SNPs. We investigated the association of rs907187, in *PARP1* promoter, and rs2048426 in non‐coding region with response chemotherapy in 121 Italian patients with high‐risk NB. Results showed that minor G allele of rs907187 associated with induction response of patients (*P* = .02) and with decrease PARP1 mRNA levels in NB cell line (*P* = .003). Furthermore, rs907187 was predicted to alter the binding site of E2F1 transcription factor. Specifically, allele G had low binding affinity with E2F1 whose expression positively correlates with PARP1 expression and associated with poor prognosis of patients with NB. By contrast, we did not find genetic association for the SNP rs2048426. These data reveal rs907187 as a novel potential risk variant associated with the failure of induction therapy for high‐risk NB.

## 1 INTRODUCTION

Neuroblastoma (NB) is the most frequent malignant tumour in paediatric age arising from neural crest cells, precursors of the sympathetic nervous system.^1^ It is an enigmatic tumour due to its high genetic heterogeneity and because of the complexity to develop a successful therapy by clinicians and researchers. The therapeutic regimens for high‐risk patients (defined following the International Neuroblastoma Risk Group (INRG) classification system^2^) include in the first phase an induction therapy with an intensive cycle of chemotherapeutic agents. Afterwards, patients follow a consolidation phase with myeloablative therapy, stem cell transplantation (SCT) and radiation therapy to primary tumour and residual metastatic sites, followed by maintenance aimed at controlling minimal residual disease.^3^, ^4^ Analyses of recent published high‐risk NB trials emphasize the large proportion of patients who do not continue beyond induction chemotherapy owing to inadequate response. For example, in the recent Children's Oncology Group (COG) trial examining purged versus non‐purged peripheral blood stem cell transplantation, 25% of the 495 registered patients did not continue to treatment beyond induction chemotherapy, most commonly because of progressive disease (56%).^5^ A larger proportion of patients (51.5% of 1,231) failed to continue beyond induction therapy in the International Society of Pediatric Oncology Europe Neuroblastoma (SIOPEN) high‐risk NBL‐1 trial.^6^ Though the rates of patients continuing to consolidation therapy differ, partly owing to differing consolidation criteria the underlying issue remains that a substantial number of patients fail to respond to current high‐risk NB induction therapy. Therefore, advanced NB remains one of the most unmanageable paediatric cancers with long‐term survival rate below 50%.^7^

The development of reliable biomarkers for clinical implementation is therefore a priority. *MYCN* amplification and segmental chromosomal aberrations are, so far, the most reliable genomic biomarkers for the patients’ stratification and outcome prediction. Recently, genome‐wide association studies and high‐throughput sequencing‐based studies have highlighted that multiple DNA polymorphisms influence NB susceptibility and clinical phenotype^8^, ^9^, ^10^, ^11^, ^12^, ^13^ and that recurrent mutations of single genes are infrequent in primary NB with activating mutations in *ALK* and inactivating mutations in *ATRX*, and *TERT* rearrangements being the most frequent.^14^, ^15^, ^16^, ^17^, ^18^ Gene expression‐based studies suggest that among high‐risk patients, gene signatures can identify children with higher risk disease who would benefit from new and more aggressive therapeutic approaches.^19^, ^20^, ^21^ Despite these large efforts made to find genomic biomarkers for improving high‐risk patient outcome, so far no study has searched for heritable variations able to predict the primary effect of chemotherapy.

One of the main reasons responsible for cancer therapeutic failure is the acquisition of resistance phenotypes to cytotoxic anticancer drugs. This is mainly because of the efficiency of the DNA repair system of cancer cells, which enhances the tolerance to DNA damages induced by chemotherapy and radiotherapy.^22^, ^23^ DNA damaging cancer therapeutics take advantage of overlapping DNA repair pathways, including base excision repair (BER), nucleotide excision repair (NER), double‐strand break repair (DSBR) and mismatch repair (MMR) pathways.^24^ As BER is one of the major DNA repair pathways, reducing BER capacity is a useful approach for cancer treatment.^25^ *PARP1* belongs to the family of the poly (adenosine diphosphate‐ribose) polymerase (PARP) proteins, which are DNA damage sensors, with the ability to signal to downstream effectors and with that directly involved in genomic stability, DNA repair and apoptosis.^24^ The roles of *PARP1* in the DNA damage response have been studied extensively. Induction of various kinds of DNA damage results in rapid recruitment of PARP1 to sites of damage through its DNA‐binding ability.^26^ It is involved in (DSBs) in both homology‐directed repair (HDR) and non‐homologous end‐joining (NHEJ) pathways. In addition, single‐strand breaks (SSBs) are very rapidly detected and bound by *PARP1*, which also represents one of the components of the BER process simplifying the subsequent recruitment of BER proteins.^27^

PARP inhibitors became interesting tools to boost the activity of cancer chemotherapy. Therefore, many clinical trials are examining their efficiency in combined therapy approaches in different sets of patients with cancer.^28^ Taking into account of the evident number of patients with NB that do not reply therapies, there is interest to early identify the non‐responder patients to allow their enrolment to suitable treatments. The purpose of our study is to identify functional single nucleotide polymorphisms (SNPs) of *PARP1* able to predict the response to current induction therapy in patients with high‐risk NB.

## 2 MATERIALS AND METHODS

### 2.1 Microarray datasets

**Figure 1:**
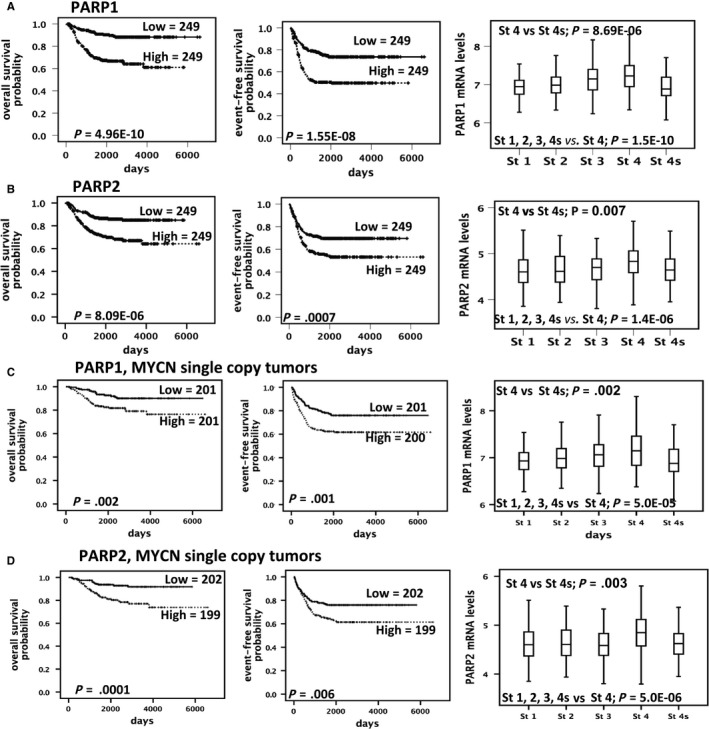
*PARP1* and *PARP2* overexpression is associated with poor survival and advanced stage in NB patients (A and B) Kaplan‐Meier analysis using published array data (dataset 1) from 498 patients and box plots showing the log2‐transformed expression profiles divided by INSS stage categories. (C and D) Kaplan‐Meier analysis using published array data from 402 patients and box plots showing the log2‐transformed expression profiles divided by INSS stage categories considering only non‐*MYCN* amplified cases

*PARP1* and *PARP2* normalized gene expression arrays of three independent sets of patients with NB were downloaded from the website R2: Genomics Analysis and Visualization Platform (http://r2.amc.nl) (Figure 1). In detail, the R2 Genomics Platform is a free, publicly accessible web‐based genomics analysis and visualization platform allowing biomedical researchers to integrate, analyse and visualize clinical and genomics data. Dataset 1) including 498 samples (among which 402 non‐*MYCN* amplified) profiled by RNAseq (http://www.ncbi.nlm.nih.gov/geo/query/acc.cgi?acc=GSE62564); dataset 2) including 88 samples (among which 72 non‐*MYCN* amplified) profiled by Affymetrix Human Genome U133 Plus 2.0 (http://www.ncbi.nlm.nih.gov/geo/query/acc.cgi?acc=GSE16476); and dataset 3) including 283 samples (among which 228 non‐*MYCN* amplified) profiled by Human Exon 1.0 ST Array (http://www.ncbi.nlm.nih.gov/geo/query/acc.cgi?acc=GSE85047). To test the association of gene expression levels with overall survival, individual gene expression profiles were dichotomized by median split into ‘high’ or ‘low’ expression groups, and Kaplan‐Meier survival curves were plotted for each group. The log‐rank test was used for comparison of survival curves. The significant difference in gene expression among the tumour stages was evaluated with Mann‐Whitney test.

### 2.2 Cataloguing of functional SNPs in *PARP1*

**Figure 2:**
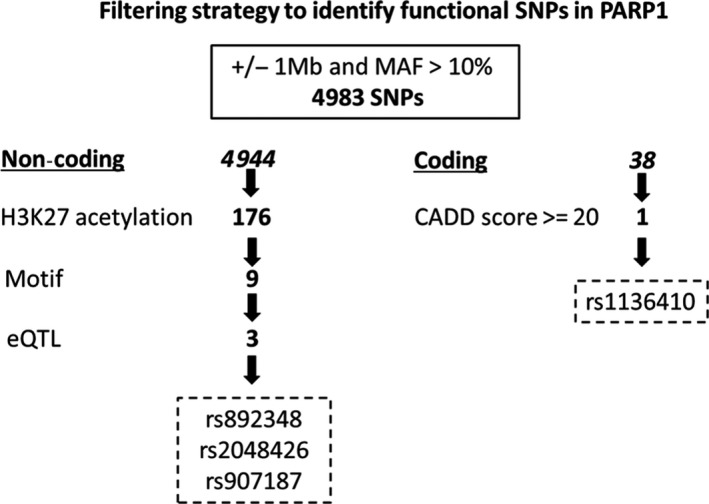
The filtering strategy of SNPs in *PARP1* to identify functional variants. Representative scheme of the filtering strategy used to identify functional SNPs in *PARP1*

To identify functional SNPs, we listed 4983 SNPs in *PARP1*, within 1 ± Mb surrounding the gene and with minor allele frequency (MAF) greater than 10%. Thus, in order to identify SNPs in *PARP1* gene that may be associated with NB patients’ induction response, we performed a filtering strategy of *PARP1* variants (Figure 2 and Table S1).

### 2.3 SNP genotyping

The SNPs rs907187 and rs2048426 were genotyped by TaqMan SNP Genotyping Assay as previously described^29^ on 7900HT Real‐time PCR System (Applied Biosystems). To monitor quality control, three DNA samples per genotype were genotyped by Sanger sequencing (3730 DNA analyzer; Applied Biosystems) and included in each 384‐well reaction plate; genotype concordance was 100%. To confirm genotypes, we sequenced 20 samples chosen randomly from responders and non‐responders; concordance between genotype was 100%. Primer sequences are available upon demand.

### 2.4 Construction of luciferase reporter gene plasmids

The genomic region of 1111 bp expanding from 555 bp upstream to 555 bp downstream the variant rs907187 was cloned upstream of the firefly luciferase gene. PCR primers contained recognition sites for NheI in the forward and XhoI in the reverse primer were designed to amplify 1153 bp from the genomic DNA of cell lines homozygous for the rs907187‐G allele. After cutting the fragment with NheI and XhoI restriction enzyme (Biolabs), we cloned it into the pGL3‐Basic Vector (Promega). The resulting plasmid containing the rs907187‐G allele was site‐specifically mutated to C alleles using Site‐Directed Mutagenesis Kit (Stratagene). The sequence of each construct was confirmed by direct sequencing.

### 2.5 In vitro functional analysis

SKNBE2 cells were transfected X‐tremeGENE (Roche) with 1 ug of pGL3‐Basic Vector rs907187‐C and rs907187‐G constructs. Cells were subsequently starved in serum‐free medium for 8 hours. Fifteen nanograms pRL‐TK Vector (Promega) was cotransfected as a normalizing control. Cells were induced to re‐enter the cell cycle by the addition of fresh medium supplemented with 10% FBS for 12 and 24 hours. At these time‐points, the cells were harvested, lysed and analysed for luciferase activity using the Dual‐Luciferase Reporter Assay System (Promega) on a TD20/20 Luminometer (Turner Designs). Results are reported as relative luciferase activities, which are obtained by dividing firefly luciferase activity with Renilla luciferase activity. Data represent the means ± Standard Deviation (SD) of three independent transfections.

### 2.6 Cell culture

The human SKNBE2, SKNFI and SKNAS cell lines were obtained from the American Type Culture Collection (respectively ATCC CRL‐2271, Cat CRL‐2142 and CRL‐2137); the human KELLY cell lines were obtained from European Collection of Authenticated Cell Cultures (92 110 411) the human MHHNB11 cell line was obtained from the Leibniz Institute DSMZ—German Collection of Microorganisms and Cell Cultures (DSMZ ACC‐157) and the human NB‐1 cell line was donated from Professor Alessandro Quattrone (University of Trento, Italy). We used the in‐house available NB cell lines with rs907187‐CC/CG/GG genotype. SKNAS and SKNFI cell lines were grown in Dulbecco's Modified Eagle's Medium (DMEM; Sigma); SKNBE2 cell line was grown in 1:1 mixture Minimal Essential Eagle's Medium (MEM; Sigma) and Nutrient Mixture F12 (Sigma); KELLY, MHHNB11 and NB1 cell lines were grown in RPMI‐1640 Media (Sigma). The medium was supplemented with 10% heat‐inactivated FBS (Sigma), 1mmol/L L‐glutamine, penicillin (100 U/mL) and streptomycin (100 mg/mL; Invitrogen). The cells were cultured at 37°C, 5% CO_2_ in a humidified atmosphere. The cell lines used for all the experiments were re‐authenticated and tested as mycoplasma‐free. Early‐passage cells were used and cumulative culture length was less than 3 months after resuscitation.

### 2.7 Western blotting

Cell pellets were resuspended and lysed in a RIPA buffer (10 mmol/L Tris‐Cl pH 8.0, 1 mmol/L EDTA, 0.5 mmol/L EGTA 1% Triton X‐100, 0.1% sodium deoxycholate, 0.1% SDS and 140 mmol/L NaCl) in the presence of a protease inhibitors cocktail (Roche). The protein concentrations were determined by Bradford assays (Bio‐Rad). Thirty micrograms of protein was loaded and separated using 10% polyacrylamide gels and transferred onto polyvinylidene difluoride membranes (Bio‐Rad). The membranes were blocked with 5% not‐fat dried milk (Sigma) in phosphate‐buffered saline (PBS) with 0.1% Tween (PBS‐T) for 1 hour and then probed with anti‐PARP‐1, (9532; Cell Signaling) antibody and E2F‐1 (sc—251 Santa Cruz Biotechnology). After a wash in PBS‐T, the membranes were incubated with horseradish peroxidase‐conjugated anti‐rabbit secondary antibody (1:4000 dilution; ImmunoReagents), and then, the positive bands were visualized using the ECL kit SuperSignal West Pico Chemiluminescent Substrate (Pierce). A mouse anti‐β‐actin antibody (1:10 000 dilution; A2228; Sigma) was used as the control for equal loading.

### 2.8 Real‐time RT‐PCR

The expression levels of *PARP1* and *PARP2* genes were analysed using quantitative real‐time PCR. Total RNA extraction of 20 stage 4 NB tumours was performed using TRIzol LS Reagent (Invitrogen) and cDNA retrotranscription using the SensiFAST cDNA Synthesis Kit (Bioline), according to the manufacturer protocol. Gene‐specific primers were designed using Primer Express 3.0 (Applied Biosystems). Real‐time PCR was performed using SensiFAST SYBR® Hi‐ROX Mix (Bioline). All real‐time PCR reactions were performed using the 7900HT Fast Real‐Time PCR System (Applied Biosystems). The experiments were carried out in triplicate for each data point. The housekeeping gene β‐actin was used as internal control. Relative gene expression was calculated using the 2^−ΔCT^ method, where the ΔCT was calculated using the differences in the mean CT between the selected genes and the internal control (β‐actin). List of Primers: *PARP1* For: AAGCCAGTTCAGGACCTCATCA, Rev: AAGGTCGATCTCATACTCCACCAT, *PARP2* For: GACGAGCTCTCATGGAAGCAT Rev: GGATTAGTGGAGGAGTACGGAGTC, β‐actin For: CGTGCTGCTGACCGAGG Rev: GAAGGTCTCAAACATGATCTGGGT.

### 2.9 NB Cell lines high‐throughput profiling

Genotyping was performed on the Illumina HumanOmniExpressExome‐8v1 BeadChip (GPL18224) (Table S6). SNP array data were processed according to the recommendations of the manufacturer. Gene expression profiles of 13 *MYCN* amplified NB cell lines were downloaded by Gene Expression Omnibus (http://www.ncbi.nlm.nih.gov/geo/query/acc.cgi?acc=GSE56552).^30^ Additional gene expression profiles of NB cell lines were obtained and processed as reported in our previous work.^30^

## 3 RESULTS

### 3.1 Association between clinical outcome and *PARP1* and *PARP2* expression in NB patients

To make evident the association of *PARP1* with clinical outcome of patients with NB, we evaluated its expression in three public gene expression datasets of NB (described in materials and methods). Data in Figure 1A; Figures S1A and S2A show high *PARP1* expression is significantly associated with low overall and event‐free survival, and unfavourable stages in dataset 1. We also tested the correlation of expression *PARP2* with survival and tumour stages and found a positive correlation in only 2 datasets with lower effect respect to *PARP1* (Figure 1B; Figures S1B and S2B). The same associations were found in non‐*MYCN* amplified tumours (Figure 1C‐D; Figures S1C‐D and S2C‐D). We also tested the association between *PARP1* and *PARP2* expression with clinical outcome by quantitative real‐time PCR (RT‐qPCR) analysis of 20 mRNA samples extracted from stage 4 neuroblastomas. We confirmed a stronger association of *PARP1* overexpression with a worst clinical outcome than *PARP2* overexpression. (Figure S3A‐D).

### 3.2 Identification of functional SNPs in *PARP1*

To define a set of credible risk variants in *PARP1* gene, that may be associated with NB patients’ induction response, we selected 176 variants (Figure 2 and Table S1) within regions of putative enhancer activity (H3K27 acetylation) in at least 15 out of 27 cell lines (25 NB and 2 neural crest cells) by using ChIPseq data deposited in GEO database (http://www.ncbi.nlm.nih.gov/geo/query/acc.cgi?acc=GSE90683). Then, in order to highlight potentially functional variants, we selected 9 variants (coloured in red in the Table S1) that altered the binding sites (prediction made by motif braker^31^) of transcription factors experimentally (ENCODE project) found to occupy the same sites (Figure 2; Table S1). As last step, among the selected 9 variants, we considered those which affect the quantitative trait loci expression (eQTLs) of PARP1 (GTEx portal data in Table S2). This resulted in 3 highly significant polymorphisms in non‐coding regions: rs892348, rs2048426 and rs907187 (coloured in yellow in the Table S1). We also selected 1 coding SNP (rs1136410) predicted highly pathogenic through CADD score (=32) (Figure 2 and Table S3). As reported in Figure S4, rs892348, rs907187 and rs1136410 are in strong LD (>0.9), so we decided to analyse rs907187, in *PARP1* promoter, and rs2048426 in non‐coding region.

### 3.3 Patient characteristics

Among the available Italian cohort of 601 patients with NB, we selected for our study 121 stage 4 patients, who undergo induction chemotherapy in the HR‐NBL‐1/SIOPEN trial (NCT01704716). The drugs used were cisplatin, vp‐16, vincristine, cyclophosphamide and either carboplatin (COJEC arm) or adriamycin (N5‐MSKCC arm). No difference in event‐free survival was observed between the two regimens (Advances in Neuroblastoma Research Association Meeting, San Francisco 2018, Abstract 90). Patients were divided into two subgroups: responders and non‐responders to induction chemotherapy, according to the definition recently reported for high‐risk patients enrolled in the SIOPEN high‐risk protocol.^6^, ^32^ Precisely, responders are the patients that can proceed with high‐dose chemotherapy and stem cell transplants, whereas non‐responders are the patients who cannot proceed and are referred to second line therapy. As reported in Table 1, response to induction chemotherapy did not associate with known prognostic markers.

**Table 1:** Patient characteristics

Characteristics	No. of patients (%)		*P*‐value
	Responders (N = 55)	Non‐responders (N = 66)	
Gender			
Male	21 (38.2%)	17 (25.8%)	
Female	34 (61.8%)	49 (74.2%)	.14
1p36 deletion			
No	23 (41.8%)	30 (45.5%)	
Yes	20 (36.3%)	27 (40.9%)	
n.a.	12 (21.8%)	9 (13.6%)	.93
Age >18 months			
No	8 (14.5%)	5 (7.6%)	
Yes	47 (85.5%)	61 (92.4%)	.217
MYC‐N amplification			
No	26 (47.3%)	41 (62.1%)	
Gain	24 (43.6%)	22 (33.3%)	
n.a.	5 (9.0%)	3 (4.5%)	.16

Abbreviation: n.a., not available.

### 3.4 Association of rs907187 G allele with response to induction therapy

In the analysis of all 121 patients, the minor G allele of rs907187 (in the promoter region of *PARP1)* associated with a better response (*P* = .02, Table 2), by contrast, we found no genetic association for the SNP rs2048426 (Table 2).

**Table 2:** Association between *PARP1* polymorphisms and response to chemotherapy

SNP	Responders	Non/Responders	*P*	OR (95% CI)
*rs907187*				
Genotypes				
CC	33 (0.60)	51 (0.81)		
CG	21 (0.38)	11 (0.17)		
GG	1 (0.02)	1 (0.02)	.02^a^	0.46
Alleles				
C	87 (0.79)	113 (0.90)		
G	23 (0.21)	13 (0.10)	.02	0.44 (0.209‐0.908)
***rs2048426***				
Genotypes				
CC	23 (0.45)	32 (0.51)		
CT	22 (0.43)	22 (0.35)		
TT	6 (0.12)	9 (0.14)	.80^a^	0.98
Alleles				
C	68 (0.67)	86 (0.68)		
T	34 (0.33)	40 (0.32)	.79	0.93 (0.533‐1.624)

Note

rs907187: genotyping failed for 3 samples; rs2048426: genotyping failed for 7 samples.

Abbreviations: CI, Confidence Interval; OR, Odds Ratio with respect to the minor (risk) allele.

a Armitrage's trend test.

The multivariate analysis including the prognostic factors *MYCN*, age at diagnosis and 1p36 deletion confirmed the association between G allele and good response to the therapy (Table S4). We also tested whether the SNP genotypes were associated with overall and event‐free survival, and prognostic factors (Figure S5 and Table S5). No significant associations were found.

**Figure 3:**
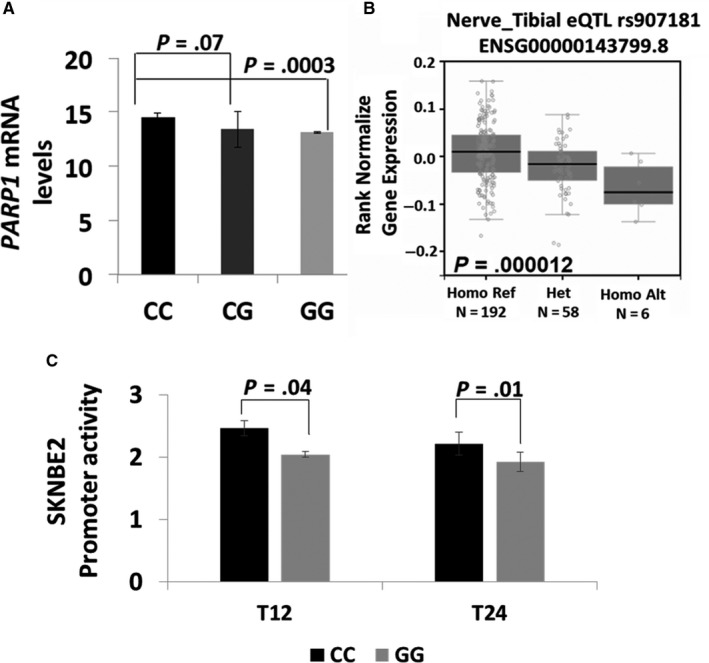
PARP1 genotype and gene expression association. (A) Microarray‐based expression profiling on 17 neuroblastoma cell lines shows a significant association between PARP1 expression and rs907187. (B) SNP‐expression correlation on Genotype‐Tissue Expression (GTEx) through the GTEx portal of Nerve Tibia Tissues. (C) The G allele of rs907187 down‐regulates promoter activity. Transcriptional activity of the pGL3‐PARP1‐CC (CC) and pGL3‐PARP1‐GG (GG) constructs in SKNBE2 neuroblastoma cells

We corroborated these data analysing the gene expression variation, using genome‐wide expression and SNP arrays of NB tumours, demonstrating that the SNP affects the expression of *PARP1*. In particular, the presence of the protective allele G correlated with decreased *PARP1* mRNA expression in a set of 17 NB cell lines (Figure 3A; *P* = .0003). This result was furthermore explored through SNP‐expression correlation on Genotype‐Tissue Expression (GTEx) project available through the GTEx portal. Notable, G allele of rs907187 correlated with a lower *PARP1* expression in the analysis of 256 Nerve Tibia Tissues (Figure 3B; *P* = .000012). Finally, to further assess the impacts of rs907187 on *PARP1* expression, we performed a luciferase report gene assay and observed that the induction of promoter activity of the construct containing rs907187‐G alleles was lower than that of the construct containing C alleles in NB SKNBE2 cells (Figure 3C). Together, these findings indicated that the decrease in *PARP1* expression because of the rs907187‐G allele may predispose NB patients to better response to induction therapy and support a potential role of *PARP1* as a candidate gene in therapeutic failure of NB treatment.

### 3.5 rs907187 is predicted to alter the binding site of *E2F1*

**Figure 4:**
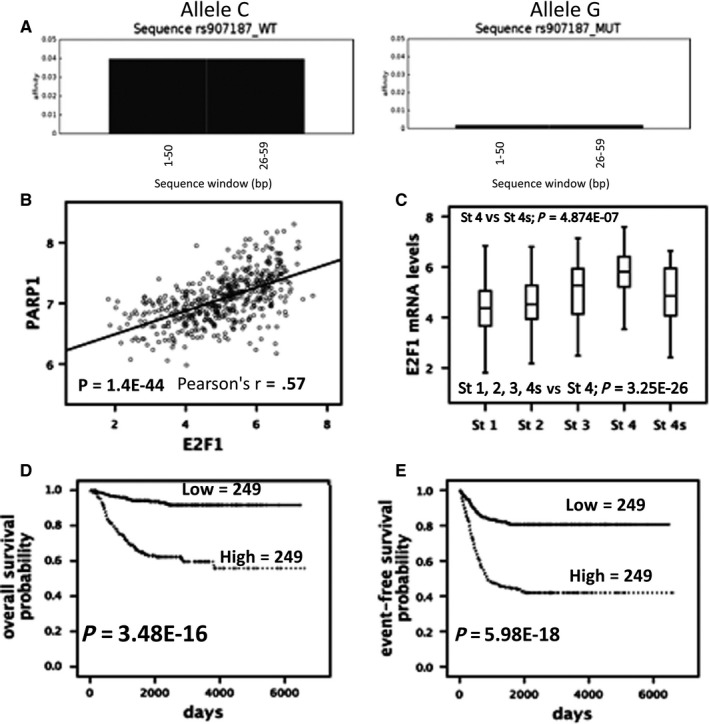
Correlation between *PARP1* and *E2F1.* (A) Prediction of nucleotide binding specificity for E2F1 transcription factor according to the rs907187 alleles (C/G). (B) Graphical representation of gene expression levels correlation between *E2F1* and *PARP1*. (C) Box plot showing the *E2F1* mRNA levels according to INSS stages using published array data from 498 patients. (D‐E) Kaplan‐Meier analysis using published array data from 498 patients

The tool TRAP^33^ predicted a high binding affinity of the risk allele C to E2F1 (E2F transcription factor 1) that was not observed with G allele (Figure 4A). The E2F1 transcription factor plays a crucial role in the control of cell cycle acting as an oncogene,^34^, ^35^, ^36^ so we tested whether its expression is correlated with the expression of *PARP1* in 498 primary neuroblastomas (http://www.ncbi.nlm.nih.gov/geo/query/acc.cgi?acc=GSE62564) and found a positive correlation between the two genes (Figure 4B). Accordingly, *E2F1* overexpression was also associated with advanced stage (Figure 4C), poor overall survival (Figure 4D) and event‐free survival (Figure 4E) in the same dataset.

We also found a positive correlation between PARP1 and E2F1 protein levels in 6 selected NB cell lines. We observed that NB cells rs907187‐CC have higher PARP1 (*P* = .0008) and E2F1 (*P* = .005) protein levels compared to rs907187‐CG/GG cells (Figure S6A‐B).

## 4 DISCUSSION

The way in which a cancer patient responds to drug treatment is dependent on many variables and among these, the individual genotype is an essential factor for influencing drug behaviour. Because of the importance of DNA repair process in determining drug sensitivity and resistance, in various cancer types, the role of polymorphisms in DNA repair genes have been explored to explain inter‐individual differences in the treatment response or survival.^37^, ^38^ Many studies focused their attention on the role of *PARP1* polymorphisms and their relation with increased risk for various cancer types.^39^, ^40^ In current literature, exist various scientific works based on the prognostic value of *PARP1* expression in human malignancies. For instance, high *PARP1* mRNA levels are found in tumours as hepatocellular carcinoma, small‐cell lung cancer and breast carcinoma.^41^, ^42^, ^43^ In NB, lncRNA FOXD3‐AS1 was reported to inhibit tumour progression through repressing PARP1‐mediated CTCF activation.^44^

In the latest years, it has been reported that polymorphisms in *PARP1* are associated with response to platinum‐based doublet chemotherapy in patients with non–small‐cell lung cancer.^45^ In addition, a recent work makes evident that SNP in *PARP1* alters the response to therapy in cancer cells.^46^ Several polymorphisms have been identified in the *PARP1* gene; the most studied is T/C‐rs1136410 resulting in a decreased enzymatic activity.^47^ Moreover, numerous studies correlate SNPs in *PARP1* with susceptibility of diverse malignancies 48, 49 and recently the PARP1 SNP rs1136410 has been associated with NB tumours arising from mediastinum in Chinese population^50^ but no association was found with the risk to develop NB.

Here, we first show that high *PARP1* mRNA levels associated with a worse outcome in three different datasets of patients with NB, suggesting that *PARP1* expression may antagonize the effect of chemotherapeutic agents used in current NB therapy. We then selected and analysed two variants in *PARP1* (rs907187 and rs2048426). Our data comprehensively reveal that the G allele of rs907187, located in promoter region, was associated with reduced activity of the dual‐luciferase in a promoter reporter, with decreased *PARP1* mRNA and protein expression in a panel of NB cell lines, and, most importantly, with response to induction therapy in our cohort of NB Italian patients. We also tested whether the SNP genotypes were associated with overall and event‐free survival, and prognostic factors but no significant associations were found. Together, these findings indicated this SNP as a novel risk variant associated with failure of current chemotherapy for high‐risk NB; though additional genetic association studies in NB cases with different ethnic origins are needed to further validate our findings. Interestingly, we provide preliminary results suggesting that the SNP rs907187 might alter the binding site of E2F1, which is a transcription factor involved in the regulation of many cellular processes including cell proliferation, DNA damage response and apoptosis.^35^ Recent evidence shows that E2F1 has a role in oxidative metabolism changing from oxidative to glycolytic metabolism under stressful conditions.^51^ E2F1 also affects migration and invasion of cancer cells and^34^ lately has been observed that it has an emerging role in melanoma executing critical functions in response to UV.^52^ DNA damage facilitates the phosphorylation and stabilization of E2F1^53^ and because PARP1 is essential for genomic stability, the link between PARP1 and E2F1 is of considerable interest. Scientific literature reports that PARP1 physically interacts with E2F1^54^ and that this interaction is increased by treatment of cells with PARP inhibitors^55^ suggesting that E2F1 activities might be regulated through PARP1 via diverse physical interactions. However, functional in vitro experiments are needed to verify if the *PARP1* SNP rs907187 alters the binding site of E2F1.

Currently, the development of PARP inhibitors has been one of the promising uncovering for cancer chemotherapy. Indeed, the United States Food and Drug Administration (FDA) approved in March 2017, niraparib for maintenance therapy of recurrent gyneacologic cancers which are sensitive to previous platinum‐based chemotherapy irrespective of BRCA mutation and homologous recombination deficiency status.^56^ So far, encouraging results have been obtained from testing PARP inhibitors in NB pre‐clinical models in drug combination schemes.^57^, ^58^, ^59^ If the genetic association, here found, will be validated in prospective studies, in future novel guidelines for the treatment of patients with NB based on their SNPs in *PARP1* might be generated. In particular, patients with rs907187‐C might be stratified and in accordance with their *PARP1* mRNA levels might be addressed towards a different therapeutic treatment with *PARP* inhibitors. This biological insight and our findings, thus, encourage the genotyping screening of patients with NB for *PARP1* variants in order to provide more efficient and personalized therapies.

## CONFLICT OF INTEREST

The authors declare no conflict of interest.

## AUTHOR CONTRIBUTIONS

MA performed the research and wrote the paper, VAL and FC analysed the data, SC, AC, AM and DC performed the research, RH, LA and AQ contributed essential reagents and tools, AG and AI designed the research study, MVC performed the research and analysed the data, and MC designed the research study and wrote the paper.

## Data Availability

The data generated and analysed during the current study are available from the corresponding author on reasonable request. Public data and data repositories are referenced within the manuscript.
