# Supplementary material for: Association of PARP1 polymorphisms with response to chemotherapy in patients with high‐risk neuroblastoma
Source: J Cell Mol Med. 2020 Feb 27;24(7):4072–81. doi: 10.1111/jcmm.15058 (PMC7171401; doi:10.1111/jcmm.15058)

**Supplementary Figure 1*.* Dataset 2*, PARP1* over-expression is associated with poor survival and advanced stage in NB patients. (A-B)** Kaplan–Meier analysis using published array data (dataset 2) from 88 patients and box plots showing the Log2 transformed expression profiles divided by INSS stage categories. (**C and D**) Kaplan–Meier analysis using published array data from 72 patients and box plots showing the Log2 transformed expression profiles divided by INSS stage categories considering only non-*MYCN* amplified cases.


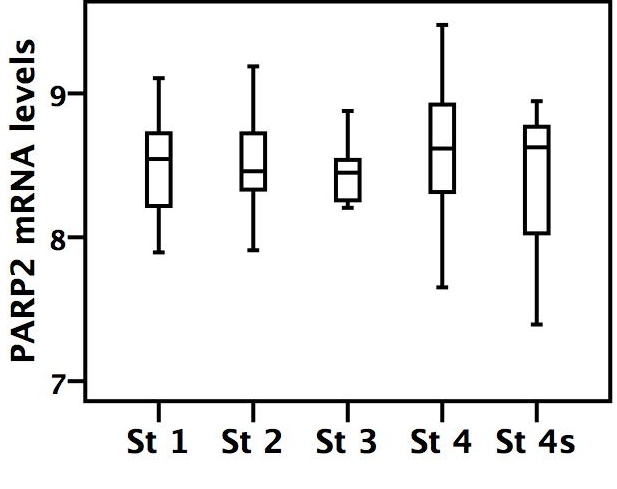


**St 4** *vs.* **St 4s; P=0.34**

**St 1, 2, 3, 4s** *vs.* **St 4; P=0.09**

**Supplementary Fig. 2. Dataset 3, *PARP1* and *PARP2* over-expression is associated with poor survival and advanced stage in NB patients.** (**A-B**) Kaplan–Meier analysis using published array data from 283 patients and box plots showing the Log2 transformed expression profiles divided by INSS stage categories. (**C -D**) Kaplan–Meier analysis using published array data from 228 patients and box plots showing the Log2 transformed expression profiles divided by INSS stage categories considering only non-*MYCN* amplified cases.

.

**Supplementary Fig. 3. qRT-PCR, *PARP1* and *PARP2* over-expression is associated with poor survival and advanced stage in NB patients.** (**A-B**) Kaplan–Meier analysis using qRT-PCR data from 20 stage 4 tumors (**C -D**) Kaplan–Meier using RT-qPCR data from 14 stage tumors without *MYCN* amplification.

**Supplementary Fig. 4. Linkage disequilibrium plot of the credible risk variants non-coding region of *PARP1***


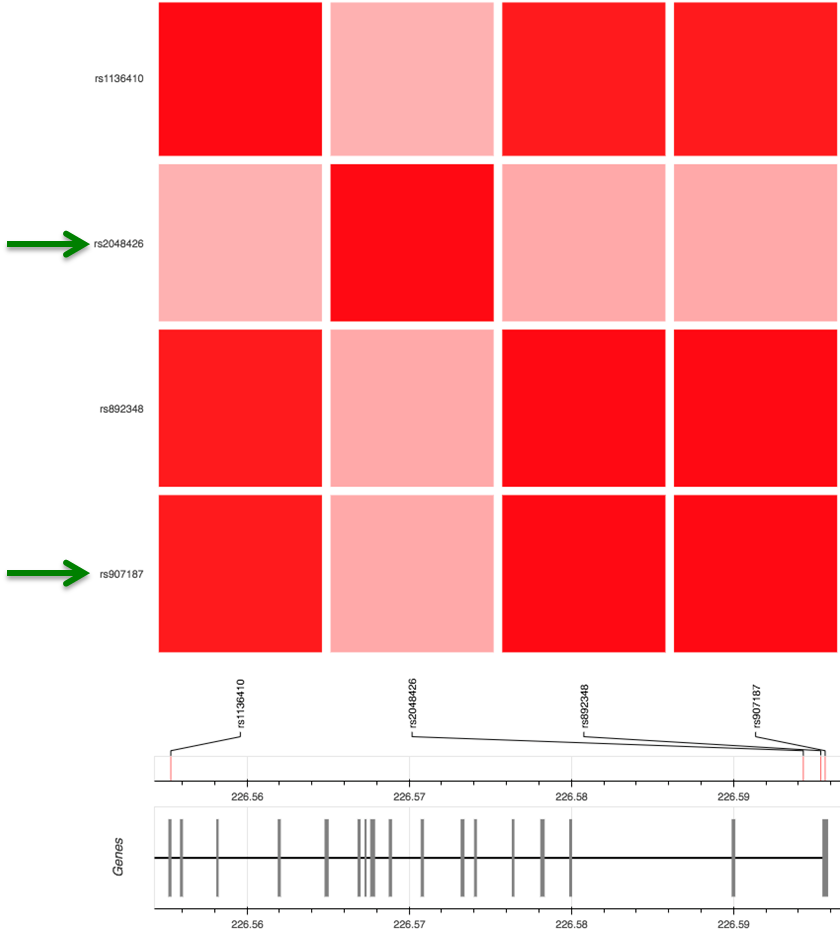


**Supplementary Fig. 5. Association between the PARP1 SNP rs907187 genotype and survival in NB patients.** **(A)** Kaplan-Meier analysis between PARP1 SNP and overall survival probability **(B)** and event-free survival probability in NB patients.


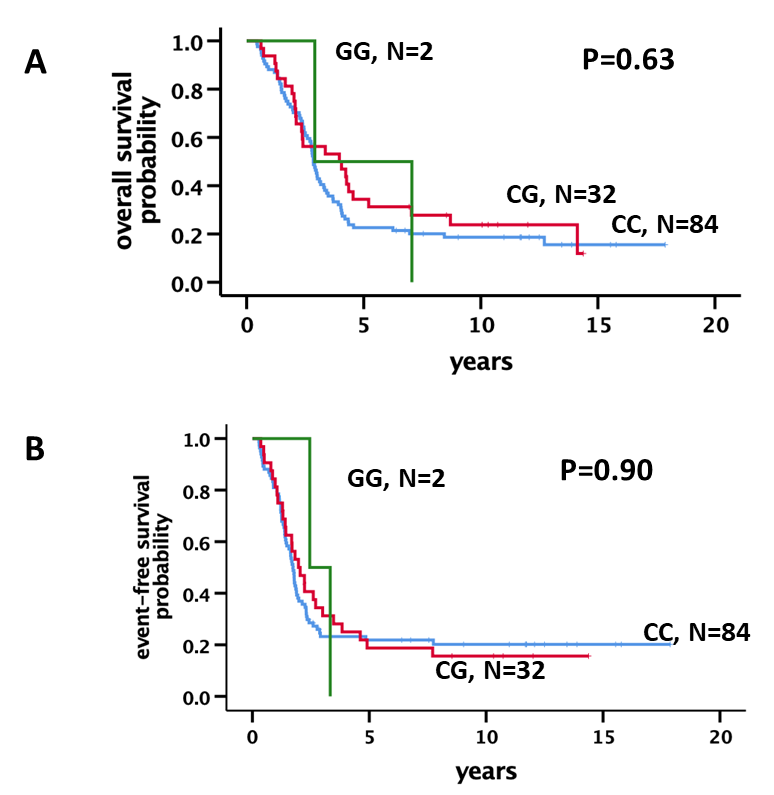


**Supplementary Fig. 6.**  **Western blot analysis of PARP1 and E2F1 proteins on NB cell lines.** (**A**) PARP1 and E2F1 protein levels were evaluated on NB cell lines (genotyped for rs907187). β-Acitn was used as the loading control. (**B**) Protein bands were quantified by densitometry. The bargraph shows the integral optic density (IOD) of average value for the bands derived from NB cell lines rs907187CC (CC) and from NB cell lines rs907187CG/GG (CG/GG), normalized respect to β-Acitn expression (B).


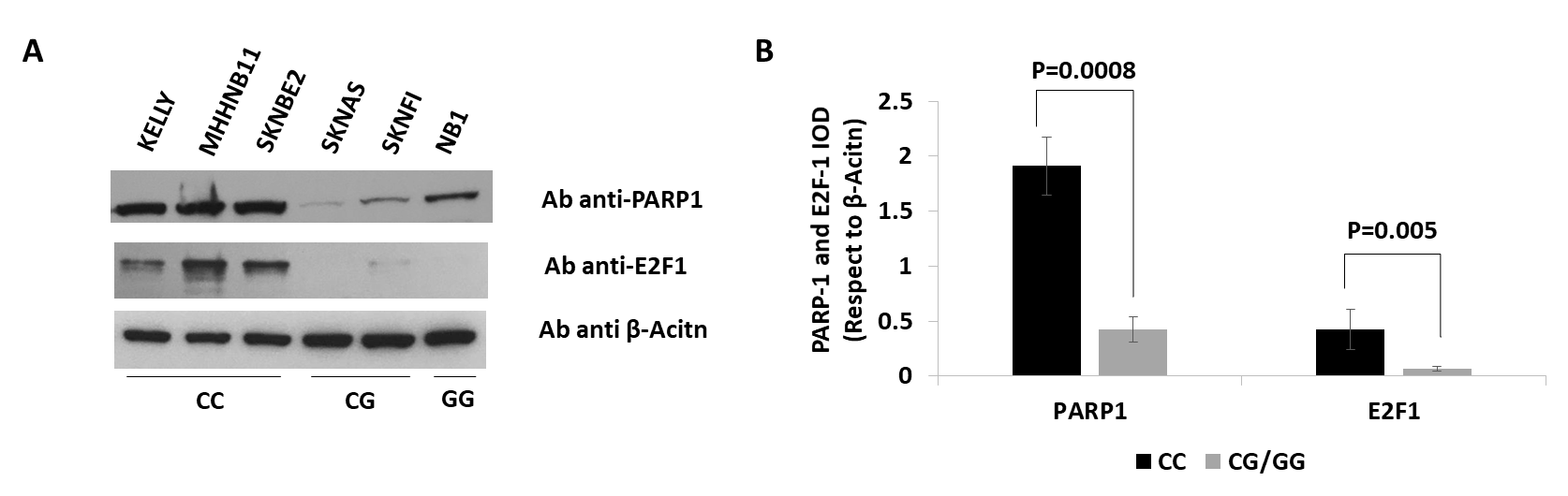

Supplement: Supplementary file 1 [file JCMM-24-4072-s001.docx]
